# The Impact of Mindfulness Programmes on Anxiety, Depression and Stress During Pregnancy: A Systematic Review and Meta-Analysis

**DOI:** 10.3390/healthcare13121378

**Published:** 2025-06-09

**Authors:** María Dolores Vázquez-Lara, Azahara Ruger-Navarrete, Samia Mohamed-Abdel-Lah, José Luis Gómez-Urquiza, Francisco Javier Fernández-Carrasco, Luciano Rodríguez-Díaz, Rafael A. Caparros-Gonzalez, Rocío Palomo-Gómez, Francisco Javier Riesco-González, Juana María Vázquez-Lara

**Affiliations:** 1 Department of Nursing, Menendez Tolosa Health Center, 11202 Algeciras, Spain; lolivazquezsas@gmail.com; 2 Department of Nursing, Faculty of Health Sciences of Ceuta, University of Granada, 51001 Ceuta, Spain; azahara.ruger@ugr.es (A.R.-N.); jlgurquiza@ugr.es (J.L.G.-U.); lucianord@ugr.es (L.R.-D.); juanamaria.vazquez@gmail.com (J.M.V.-L.); 3 Instituto Nacional de Gestión Sanitaria, 51003 Ceuta, Spain; samiadrismohamed@gmail.com; 4 Department of Nursing, Faculty of Health Science, University of Granada, 18016 Granada, Spain; rcg477@ugr.es; 5 Instituto de Investigacion Biosanitaria ibs.GRANADA, 18012 Granada, Spain; 6 Department of Obstetrics, Hospital of La Línea de la Concepción, 11300 Cadiz, Spain; rociopalomogomez@hotmail.es; 7 Department of Obstetrics, Hospital Universitario Punta de Europa, 11207 Algeciras, Spain; javieriesco75@gmail.com

**Keywords:** pregnancy, mindfulness, psychological stress, depression, anxiety

## Abstract

Background: Mental health problems that can appear in women during pregnancy include fear, anxiety, feelings of vulnerability, stress, and depression. Mindfulness (MF) is a specific meditation technique that can help during treatment for prenatal mood disorders, emotional distress, and psychological strains. The aim of this study is to determine the effectiveness of a specific meditation approach in women during pregnancy on these mental health problems. Methods: This systematic review analysed data from PubMed, Scopus, and CINAHL. The search equation used was “mindfulness [title] AND pregnancy [title] AND (trial OR clinical trial OR RCT OR quasi-experimental OR experimental OR randomised clinical trial OR randomised controlled trial OR quasi-experimental study)”. This analyses experimental studies published in the last 10 years that include interventions based on MF, applying cognitive behavioural therapies to reduce stress, depression, and anxiety and in which the participants completed a questionnaire related to these variables. Standardised means effect size meta-analysis was performed with RevMan Web. Results: All the included studies (n = 13) reported that the intervention led to a decrease in negative symptoms related to prenatal pressure, apprehension, and melancholy. The duration of the MF programmes was 6 to 8 weeks. The meta-analysis showed that MF during pregnancy is an effective approach, with a standardised mean difference of −0.73 for anxiety, −0.67 for depression, and −0.74 for stress. Conclusions: Mindfulness programmes during pregnancy are a useful and effective means of reducing maternal stress, anxiety, and depression. Including MF programmes during pregnancy should be considered depending on resources availability. In person vs. online effectiveness should be investigated.

## 1. Introduction

Pregnancy, or gestation, is the biological process that takes place in a woman from the implantation of a fertilised egg in the uterus until childbirth, during which time both physiological and psychological changes take place [1]. The emotional changes that the mother experiences will vary during the pregnancy and may continue through the postpartum period. Among the psychological alterations that can appear are fear, anxiety, and feelings of vulnerability, stress, or depression [2].

Pregnancy triggers neuroendocrine, cardiovascular, and immunological changes that collectively create a psychological “stress test” impacting maternal mental health. Both her own health and that of the foetus are influenced by this psychological stress, which can be defined as the imbalance experienced when the mother feels she cannot cope with the demands of pregnancy, an imbalance that becomes apparent at both the physiological and the behavioural level [3].

In 2019, according to the World Health Organization, about 280 million people (5% of all adults) suffered depression. The most widely used diagnostic criteria for depression, in both clinical practice and research, are those provided by the International Statistical Classification of Diseases and Related Health Problems (ICD) and the Diagnostic and Statistical Manual of Mental Disorders (DSM) [4]. In clinical and research settings, postpartum depression is typically defined as the presence of depressive symptoms occurring within 12 months after childbirth, rather than adhering to the narrower DSM-5 criteria [5]. Its emotional consequences have a unique characteristic: it can affect not only the mother but also the child emotionally, impacting attachment and mother–child bonding. Typically, it emerges during the first month postpartum, peaking in intensity between 8 and 12 weeks after birth [6]. Studies report that over 10% of women experience depression during pregnancy and/or shortly after giving birth [7]. In Spain, an estimated 10–15% of women develop depressive symptoms between the fourth and eighth week postpartum, with high-risk women showing incidence rates of 25–35% [8]. The condition of depression can last most or all of the day, almost every day, and for at least two weeks. During this time, the person affected may suffer sleep disorders, decreased appetite, weight loss, anhedonia, and reduced self-esteem, together with constant thoughts about death and hopelessness. In addition, she may feel tired and have difficulty concentrating [7,9].

Anxiety is the state of discomfort, tension, restlessness, and alarm, from a subjective perspective, that disquiets an individual. This emotion arises when individuals perceive threats, whether external or internal. In this situation, the sympathetic system is activated and adrenaline is secreted, generating the typical signs of anxiety [10]. Pregnant women are more likely to be exposed to physiological stress, such as anxiety about their babies and their new lifestyle [11]. They take on multiple roles, including the novel and unfamiliar role of motherhood, which can lead to emotional overload. An estimated 4% of the global population suffers from an anxiety disorder, making it the most common mental health disorder worldwide [12]. Pregnancy-related anxiety (sometimes referred to as prenatal or perinatal anxiety) is an intense state of distress experienced by some pregnant women, specifically focused on their condition. It may manifest as persistent fears about the baby’s health, anxiety about childbirth, distress over potential complications, or even doubts about one’s ability to mother. Unlike other anxiety disorders, it is not classified as an independent category in diagnostic manuals like the DSM-5. However, numerous studies highlight its significance, as it is associated with a 50–60% increased risk of postpartum depression and may lead to medical complications during pregnancy [13]. The Pregnancy-Related Anxiety Scale (PRA) measures general pregnancy-associated anxiety. This self-administered questionnaire assesses specific concerns, including fear of childbirth, foetal health, physical changes, and adjustment to motherhood. Responses are typically scored on a Likert scale (e.g., 1—“not at all” to 5—“very much”), with the total score indicating anxiety severity. The PRA helps identify risks for obstetric complications or postpartum depression. Its design specifically distinguishes pregnancy-related anxiety from generalised anxiety disorders, providing a targeted tool for perinatal mental health assessment [14]. Generalised anxiety disorder (GAD) is characterised by excessive and persistent worry about multiple life areas (such as health, work, family, or finances) lasting at least six months. Individuals with GAD experience both physical and psychological symptoms, including restlessness, fatigue, difficulty concentrating, muscle tension, irritability, and sleep disturbances. Unlike perinatal distress, which specifically affects women during the perinatal period, GAD is not linked to any particular life stage and can affect both men and women at any point in their lives [5].

Stress represents the body’s natural physiological and psychological response to situations perceived as overwhelming or threatening. This alarm mechanism activates systems including the hypothalamic–pituitary–adrenal (HPA) axis and sympathetic nervous system, triggering the release of hormones like cortisol and adrenaline that prepare the organism to respond to perceived danger. The World Health Organization defines stress as a series of physiological changes that prompt action. However, when this response becomes prolonged, it ceases to be adaptive and may impair both physical and emotional health. Eustress (positive stress) serves as a performance-enhancing motivator, while distress (negative stress) occurs when pressure is chronic and intense, ultimately diminishing quality of life [15].

Multiple factors contribute to these emotional alterations. These include extreme maternal age (adolescence or advanced), pre-existing conditions like neurosis or psychosis, and personal/family history of depression or suicide attempts. Additionally, psychosocial factors play a role: negative attitudes toward pregnancy, parental conflicts, lack of partner support, or traumatic life events [16].

Depression in pregnancy is known to increase the risk of preterm labour and pre-eclampsia. Moreover, it can inhibit the monitoring and control of the pregnancy, deteriorate self-care attention, and even result in suicide attempts. Furthermore, maternal depression may alter foetal neurodevelopment, resulting in a low Apgar score. Schoolchildren whose mothers suffered depression during pregnancy are more likely to present aggressive behaviour and suffer learning difficulties [17]. Likewise, pregnant women with prenatal depressive symptoms are at risk of having problems in the social sphere, emotional isolation, and excessive concern about their ability to perform their maternal role in the future [18].

The assessment of perinatal anxiety and depression is highly relevant due to their frequent comorbidity and the significant impact they can have on both the mother and her offspring. These conditions have been associated with long-term consequences in children, such as symptoms of depression and anxiety at 14 to 15 years of age, as well as attention-deficit/hyperactivity disorder (ADHD) between the ages of 8 and 9 [19].

For depression during pregnancy, two main treatments exist: psychotherapy (including relaxation techniques, cognitive behavioural therapy, and supportive therapy) and pharmacotherapy (such as antidepressants). If the depressive condition is mild, psychotherapy is the first-choice treatment but, when it is moderate to severe, pharmacological treatment is normally called for [20]. In low- and middle-income countries, health personnel may supply psychotherapy, and this type of intervention has proven to be quite effective [21].

A novel proposal for the prevention and treatment of mental health problems such as depression is that of mindfulness (MF). This is a meditation technique that has been defined as the awareness that emerges through paying deliberate attention, at the present moment and nonjudgmentally, to the unfolding of experience moment by moment. MF encourages the individual to identify negative moments in order to better tolerate them; it generates a cognitive change in the relationships between thoughts and offers cognitive experiences that foster greater understanding of the development and course of the ideas that come to mind [22].

MF offers various benefits, such as improved physical, mental, and emotional health, an enhanced ability to emotionally manage complicated situations, expanded memory capacity, reduced insomnia, and the promotion of self-knowledge [23]. A specific meditation approach is becoming increasingly better known and, in some cases, it is presented as a treatment option for women experiencing high levels of psychological distress during pregnancy [24].

The importance of this work lies, on the one hand, in the health relevance of depression as a serious mood disorder, as well as the clinical entities anxiety and stress, and, on the other hand, in that all of them are associated with a range of negative effects for mothers, infants, family members, and wider society [25]. All this determines the need to explore new therapies that mitigate the adverse results mentioned; that is why the aim of this systematic review and meta-analysis is to consider the effectiveness of mindfulness (MF) interventions in reducing levels of depression, anxiety, and stress during pregnancy.

The study hypothesis is that present-moment awareness techniques will be positive in reducing mood disturbances during pregnancy.

## 2. Methods

In conducting this systematic review with meta-analysis, the PRISMA recommendations [26] were followed at all times.

The research protocol has been registered in PROSPERO with ID 1047367.

### 2.1. Eligibility Criteria

We included primary experimental studies (randomised clinical trials or quasi-experimental designs). These studies had to:

1. Implement a mindfulness (MF) intervention during pregnancy;
2. Measure its effects on anxiety, depression, or stress post-intervention.

Other criteria applied were that the papers should have been published in English or Spanish within the last 10 years (2014 until now) and in peer-reviewed journals. Studies with mixed samples, with women before or after pregnancy, that did not report independent data for pregnancy were excluded.

### 2.2. Information Sources and Search Strategy

The PubMed, Scopus, and CINAHL databases were consulted using the following search equation, based on MeSH terms: “mindfulness [title] AND pregnancy [title] AND (trial OR clinical trial OR RCT OR quasi-experimental OR experimental OR randomised clinical trial OR randomised controlled trial OR quasi-experimental study)”. No filter or limitation was imposed on the search results. The search was carried out in March 2024.

### 2.3. Selection of Studies for Analysis

Two researchers independently screened studies, with third-party arbitration for disagreements. First, the reference manager Zotero was applied to eliminate duplicate studies. The selection was further refined by reading first the titles and abstracts and then the full texts of those remaining. Among the papers resulting from this selection, backward and forward searches were then performed of the bibliography.

### 2.4. Data Extraction and Formulation

We entered the extracted data into a table with the following variables: author(s), year and country of publication, study design, size and mean age of the population sample, description of the MF intervention, and pre- and post-intervention mean values obtained.

### 2.5. Risk of Bias and Level of Evidence

For each paper, we determined the recommendation level and evidence grade in accordance with the Oxford Centre for Evidence-Based Medicine ranking system [27]. In the critical reading process, five questions from the Mixed Methods Appraisal Tool (MMAT) checklist (2018 version) were considered for clinical trials or quasi-experimental studies, depending on the study [28], once both screening questions were answered affirmatively. We excluded studies with <2/5 positive MMAT responses. In all the studies analysed, both screening questions received a “yes” response, indicating they were suitable for evaluation with MMAT. Of the 13 studies included, 6 received a “yes” on all five questions. Five studies had four “yes” responses and one “can’t tell”, while the remaining two studies received three “yes” responses and two “can’t tell”. The individual results for each study, according to the MMAT criteria, are detailed in Appendix A.

### 2.6. Effect Measures and Data Synthesis

Three random-effects meta-analyses were performed, using RevMan Web software. The effect size for each study variable was calculated according to the post-intervention standardised mean difference, using the means and standard deviations for the levels of anxiety, depression, and stress recorded for the intervention (MF) group and the control group. Heterogeneity was calculated from the I2 value, and the presence or otherwise of publication bias was determined by Egger’s test. A sensitivity analysis was performed to assess whether any study significantly modified the effect size.

## 3. Results

### 3.1. Search Results

**Figure 1:**
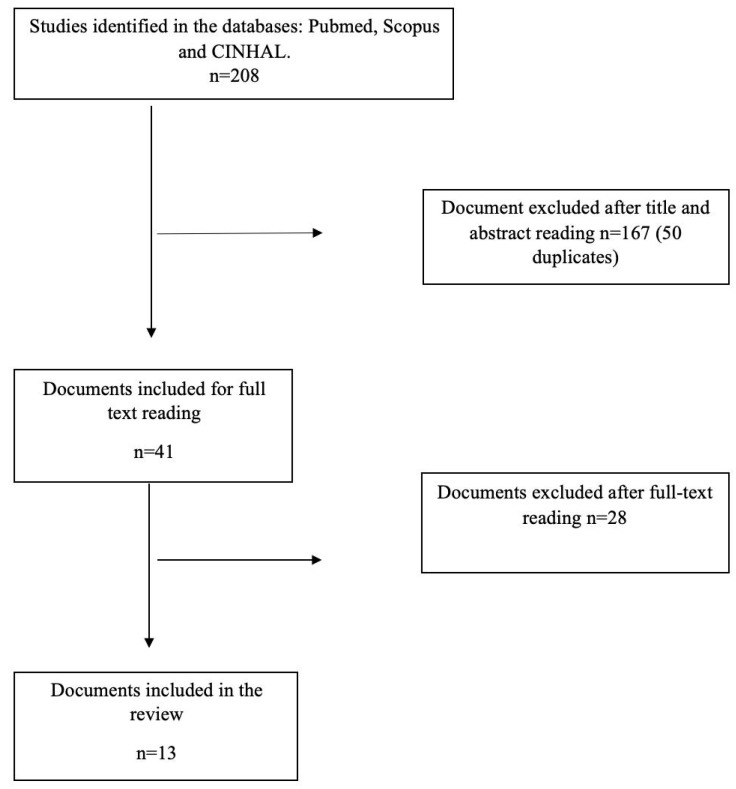
Flow chart for document selection.

The search process obtained the following results: 68 articles were recovered by Scopus, 37 by PubMed, and 103 by CINAHL. In total, 208 articles were retrieved but 50 were duplicates and hence excluded, leaving 158. The initial reading of the titles and abstracts led to a further 117 articles being discarded. The 41 articles remaining all met the inclusion criteria and focused on anxiety, depression, or/and stress during pregnancy and were selected for the full-text review (studies were excluded due to language, not measuring psychological distress, or not being experimental studies). Finally, 13 articles [29,30,31,32,33,34,35,36,37,38,39,40,41] were found to be suitable for the purposes of this analysis (see Figure 1).

### 3.2. Characteristics of the Studies Included

The studies included in the review were published between 2014 and 2023, and 10 of the 13 (77%) have appeared during the last five years. The geographic distribution was as follows: six were published in the United States, two in Iran, two in China, and one each in Sri Lanka, Indonesia, and Taiwan (Table 1).

Regarding the methodological design, of the thirteen studies analysed, eleven were randomised clinical trials [31,32,33,35,37,38,39,40,41] and two were quasi-experimental or nonrandomised pilot studies [29,30]. The latter were characterised by using small samples (n = 12 to 35 women) and by applying the mindfulness intervention to the entire sample without a control group or random assignment. Specifically, the studies by Abatemarco et al. [29] and Agampodi et al. [30] did not present a controlled design or randomisation, while Goodman et al. [34] and Kalmbach et al. [36], although without a traditional control group, were considered experimental designs, so they were methodologically treated as pilot clinical trials or proof of concept. In general terms, it can be stated that 85% of the included studies (11 out of 13) correspond to randomised designs, while the remaining 15% present quasi-experimental designs or without formal randomisation (Table 1).

The sample size varied significantly across studies (12–215 women). All participants were at risk of depression, stress, or anxiety and underwent MF interventions during pregnancy. To determine whether participants were at risk of developing such psychological distress, each of the included studies used validated mental health measurement instruments for pregnant women. This risk was established based on high scores on questionnaires such as the Edinburgh Postnatal Depression Scale (EPDS), the Generalized Anxiety Disorder-7 (GAD-7), the Pregnancy-Related Anxiety Scale (PRA), or the Depression, Anxiety and Stress Scale (DASS-21), among others. Therefore, although some participants did not have a formal clinical diagnosis, they were considered at risk due to presenting subclinical symptoms or high levels of psychological distress, which justified preventive intervention using mindfulness.

In most of the studies considered [29,30,31,32,33,34,35,36,37,38,39,40,41], the women included in these MF interventions, in which cognitive behavioural therapies were employed to reduce stress, depression, and anxiety, were aged between 18 and 45 years. In almost all of the articles analysed, two study groups were created: an intervention group, which took part in meditative practices, and a control group, which might have taken part in another type of intervention but not MF [29,30,31,32,33,34,35,36,37,38,39,40,41]. However, in four papers [29,30,34,36], the study population was very small, with only 12 to 35 participants, and the intervention was applied to the entire sample, without distinction.

Some studies developed or adapted a structured and specifically named programme, rather than applying a generic mindfulness protocol, such as Perinatal Understanding of Mindful Awareness for Sleep (PUMAS) in the Kalmbach study [36], aimed at treating insomnia and cognitive activation in pregnancy, and CALM Pregnancy (Coping with Anxiety through Living Mindfully) in the Goodman study [34], focused on managing generalised anxiety in pregnant women. These programmes differ from the rest of the studies that applied mindfulness interventions based on MBSR or standard MBCT (Table 1).

Goodman et al. [34] developed the Coping with Anxiety through Living Mindfully (CALM) intervention, an adaptation of mindfulness-based cognitive therapy (MBCT) specifically tailored for pregnant women experiencing anxiety. The programme trains participants to modify maladaptive responses to psychological distress through integrated mindfulness meditation practices, cognitive restructuring techniques, and psychoeducation about anxiety and mood disorders. A distinctive component involves self-compassion meditation, which has shown significant associations with reduced affective symptoms and enhanced psychological well-being in perinatal populations [34]. The intervention uniquely incorporates present-moment awareness exercises focused on foetal connection during both guided group sessions and home practice. Standard sessions feature didactic instruction, cognitive skill-building exercises, formal meditation training, and facilitator-led group discussions to reinforce learning.

In a related development, Kalmbach et al. [36] designed the Perinatal Understanding of Mindful Awareness for Sleep (PUMAS) programme, which adapts behavioural sleep strategies within a mindfulness-based intervention framework for pregnancy. Treatment efficacy was evaluated through pre–post assessment of patient-reported outcomes, including insomnia severity (Insomnia Severity Index), depressive symptoms (Edinburgh Postnatal Depression Scale), nocturnal cognitive arousal (Pre-Sleep Arousal Scale cognitive subscale), pregnancy-specific nighttime rumination, and sleep-related effort (Glasgow Sleep Effort Scale). This approach combines evidence-based sleep intervention components with mindfulness techniques modified for perinatal physiological and psychological needs [36]. These measures were assessed at baseline/pre-treatment and post-treatment.

All these articles described very similar structures for the MF intervention, with 6–8 weekly sessions lasting 2–3 h on average. Research conclusions were drawn from participants’ responses to a questionnaire focused on the study variable of interest, namely stress, anxiety, and/or depression during pregnancy. These questionnaires included the Pregnancy Worries and Stress Questionnaire (PSWQ) (Penn State Worry Questionnaire), Spielberger’s Trait Anxiety Inventory (STAI), the Edinburgh Postpartum Depression Scale (EPDS), the Generalised Anxiety Disorder Scale (GAD-7), the Perceived Stress Scale (PSS), the Pregnancy-Specific Anxiety Scale (PSA), the Five-Facet Mindfulness Questionnaire (FFMQ), the Beck Anxiety Inventory (BAI), the Beck Depression Inventory—Second Edition (BDI-II), the Depression, Anxiety and Stress Scale (DASS-21), the Postpartum Bonding Questionnaire (PBQ), and the Pregnancy-Related Thoughts Scale (PRT). However, two studies [40,41] described an intervention based on digital guided self-help, consisting of short video modules that participants watched via a WeChat program, after which they performed MF practices at home.

As concerns the overall results obtained, each of the papers reported a decrease (i.e., an improvement) in negative symptoms related to stress, anxiety, and depression during the perinatal period after the intervention, in comparison with the pre-intervention value obtained.

### 3.3. Results of MF in Anxiety, Stress, and Depression

Anxiety. Numerous studies indicated substantial improvements in symptoms across varied interventions. Goodman et al. [34] reported that 82% of participants initially in the clinical range for excessive worry experienced reliable improvement after receiving treatment. Kundarti et al. [37] highlighted a significant difference in mean anxiety scores between intervention (12.83 ± 8.29) and control groups (30.69 ± 16.39) with *p* < 0.000, showcasing the intervention’s efficacy. Nejad et al. [34] observed a notable decrease in mean scores for this disorder among intervention participants, while the control group exhibited no such changes. Abatemarco et al. and Zhang et al. [29,40] contributed further evidence, showing lower distress levels and significant differences in outcomes between their respective intervention and control groups, with effect sizes ranging from 0.39 to 0.85 (Cohen d).

Stress. Regarding this concept, significant reductions were observed in several interventions. Abatemarco et al. reported that Pregnancy-Specific Anxiety Scale scores decreased significantly from baseline to Post-I (*p* = 0.007) and Post-II (*p* < 0.0001), indicating the positive impact of their approach. Similarly, EPEL et al. found that women participating in Mindful Mother Training (MMT) experienced significant reductions in perceived stress (β = −0.16) compared to those undergoing usual care. Guardino et al. [35] revealed that a mindfulness intervention led to a greater decline in Pregnancy-Specific Anxiety (PSA) and Pregnancy-Related Anxiety (PAR) from baseline to post-intervention when compared to a reading-based control group, although the changes were not statistically distinct. Pan et al. further confirmed that prenatal awareness-based techniques yielded lower levels of prenatal and postnatal stress compared to control conditions. Lastly, Zhang et al. [41] demonstrated that participants in an intervention group experienced greater improvements in perceived stress (Wald χ^2^ = 26.94, *p* < 0.001) relative to their control counterparts.

Depression outcomes also reflected meaningful progress. Dimidjian et al. [32] revealed lower rates of perinatal depression relapse under Mindfulness-Based Cognitive Therapy (MBCT) (39.50%) compared to conventional treatments (63.50%). Epel et al. [33] indicated reductions in depressive symptoms (β = −0.21) for women in the MMT programme. Goodman et al. [34] noted that 69.6% of participants within the clinical depression range demonstrated significant recovery following intervention. Kalmbach et al. [36] reported substantial decreases in Edinburgh Postnatal Depression Scale (EPDS) scores pre- and post-PUMAS intervention, from an average of 8.67 ± 5.33 to 3.42 ± 2.75, corresponding to a mean reduction of 5.25 points (t [11] = −4.16, *p* = 0.002; Cohen’s dz = 1.41). Additionally, Nejad et al. and Pan et al. [38,39] highlighted significant decreases in depression scores for intervention participants relative to baseline, whereas the control group did not exhibit comparable improvements. Finally, Zhang et al. [40] observed lower depressive symptoms in a digital GSH-MBI intervention group than in controls, with Cohen d values ranging from 0.49 to 0.84.

### 3.4. Results of the Meta-Analysis

We based the meta-analyses on six studies for anxiety, five for depression, and five for stress. The intervention groups consisted of 308, 294, and 251 women, respectively, while the control groups were formed with 301, 295, and 237 women, respectively. Although one of the included studies in the meta-analysis was not randomised [33,37] in the sensitivity analysis, when the study was removed from the analysis, there were no significant changes in the effect size.

Egger’s test did not reveal publication bias in any of the meta-analyses (*p* > 0.05) and the I2 values obtained for heterogeneity were 90% for anxiety, 73% for depression, and 87% for stress.

**Figure 2:**
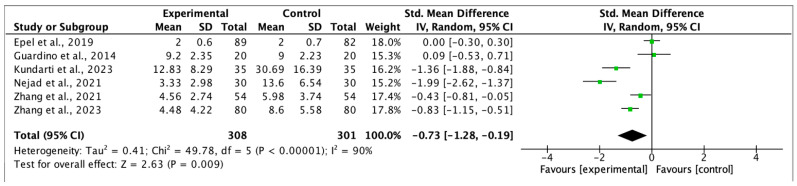
Forest plot for anxiety post-intervention [33,35,37,38,40,41].

**Figure 3:**
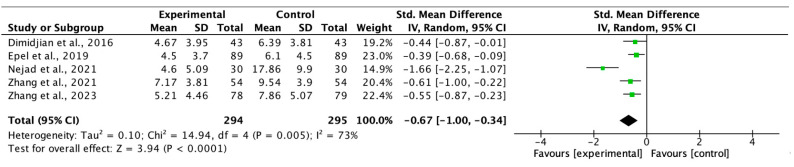
Forest plot for depression post-intervention [32,33,38,40,41].

**Figure 4:**
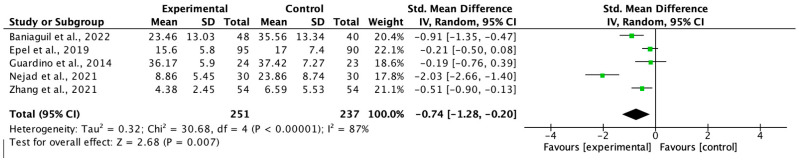
Forest plot for stress post-intervention [31,33,35,38,41].

The effect size (standardised mean difference) for the effectiveness of MF during pregnancy was −0.73 (95%CI −1.28, −0.19 *p* < 0.05) for anxiety, −0.67 (95%CI −1.00, −0.34 *p* < 0.05) for depression, and −0.74 (95%CI −1.28, −0.20) for stress, in favour of the intervention group in every case. The respective forest plots are shown in Figure 2, Figure 3 and Figure 4.

## 4. Discussion

Different investigations highlight the beneficial effects of mindfulness interventions in the three areas, depression, anxiety, and stress during pregnancy, including some of them also in the postpartum period [42,43]. In the same line of research, in another study whose objective was to evaluate the effectiveness of nondrug interventions, such as cognitive behavioural therapy (CBT), meta-analysis showed that CBT could effectively alleviate depressive symptoms in perinatal women [44].

The results found related to depression levels indicate that they decreased after the implementation of the programme; also, other research concludes that the practice of mindfulness meditation during pregnancy can help reduce stress and depression in pregnant women [45]; likewise, reviews of several studies have shown that mindfulness-based interventions (MBI) used during the perinatal period have the ability to alleviate anxious and/or depressive symptoms [6]. While some authors conclude that participation in MF programmes can alleviate these problems, others go further, claiming that these programmes can help not only reduce but prevent depression in pregnancy and that this approach could be a valuable complementary measure in clinical treatment [46].

In the same way, the symptoms of anxiety were less acute after a course of MF sessions. In accordance with these findings, other studies, too, have reported the effectiveness of the MF approach in reducing anxiety among pregnant women [47,48].

Over-reactivity to stress can provoke undesirable health outcomes, which may be particularly harmful during pregnancy. According to our analysis, MF-based interventions generate significant improvements in two important indicators of mental health, namely psychological stress and depression. Other research confirms that women who achieve greater mindfulness have significantly lower reactivity to perceived stress, which suggests that present-moment awareness may be an important protective factor in this context [49,50].

The present review is subject to certain limitations. Firstly, the studies considered are heterogeneous, relatively few, and, in some cases, based on a very small study population. The significant heterogeneity observed in the anxiety, stress, and depression outcomes likely stems from variations in participant characteristics, differences in intervention protocols, and diverse study contexts across the included research. Regarding the population, women with different gestational ages were included, ranging from the 10th to the 32nd week. Additionally, while some studies did not report the number of previous pregnancies, one required participants to be primiparous women [37]. Moreover, whereas some studies focused on women in good health [32,37,39,41], others focused on pregnant women with high levels of stress, anxiety, depressive symptoms, or other health issues [29,35,40]. There was also disparity in the socioeconomic and educational background of participants. Some studies required a university-level education or higher [35,39], while others only required participants to be literate in the language of the area where the research was conducted [37]. These differences could influence access to and participation in the interventions, thereby affecting the results. As for the interventions, although all were conducted in person by trained staff and ranged between six and eight sessions lasting 2 to 3 h each, some were complemented with the use of digital and web-based resources [39,41]. In summary, the diversity in inclusion criteria, the varying characteristics of the populations, and the interventions may explain the high heterogeneity found in the analyses of anxiety, depression, and stress, thus influencing the results.

Also, some studies did not use statistical analysis. Furthermore, perinatal care is addressed quite differently in different countries and continents, and the characteristics of their health systems vary widely.

In future research, it would be useful to investigate this issue more broadly, with additional experimental studies, to learn more about the effects of MF therapy during pregnancy. Moreover, this analysis might be extended to span the periods of childbirth and postpartum. Another valuable approach would be to make the research conclusions and results more widely known, for example, among health personnel in the obstetric field and in primary care attention (midwives and nurses) to ensure these workers receive the corresponding training and are equipped to apply the technique. In countries like Spain, this intervention could be implemented in primary care centres, as midwives offer free childbirth preparation classes at all of these centres, which pregnant women and their partners can attend at no cost. In other countries with low incomes or weakened healthcare systems, healthcare personnel could be trained to perform the present-moment awareness technique online so that it would not require in-person training and, thus, reduce the cost of training.

There is solid evidence for the practice of MF during pregnancy. Therefore, and given its low-risk nature, all women should be encouraged to consider embodied attention practices during their pregnancy [42]. Indeed, it has been shown that pregnant women seeking routine prenatal care significantly prefer nonpharmacological psychosocial treatments that include MF for the prevention of depression [51].

In the document “WHO Recommendations: Intrapartum Care for a Positive Childbirth Experience,” the World Health Organization advocates for the use of mindfulness practices alongside respectful maternity care and emotional support, as these approaches have demonstrated efficacy in alleviating fear, anxiety, and stress characteristic of the peripartum period [52].

Although this review includes studies published up to 2023, it is important to note that more recent research highlights the benefits of mindfulness during pregnancy. For instance, Feli et al. found that a brief mindfulness-based intervention significantly reduced anxiety levels in first-time mothers [53]. Meanwhile, the randomised controlled trial conducted by Gökbulut et al. directly compared a mindfulness programme (MBSR) with deep relaxation exercises, demonstrating that both interventions helped reduce pregnancy-related anxiety, with slightly better outcomes in the mindfulness group [54].

While these studies employed slightly different approaches, they support our meta-analysis findings that mindfulness therapy is a viable tool for managing stress and anxiety during pregnancy. Future research could explore whether these interventions remain effective across diverse cultural and socioeconomic populations or whether their benefits persist postpartum.

## 5. Conclusions

This research significantly supports the investigation of nonpharmacological interventions for addressing prenatal depression, as well as stress and anxiety, demonstrating that mindfulness contributes to the improvement of their symptoms. This provides a foundation for developing interventions targeting prenatal depression.

According to the results obtained by this study, prenatal mindfulness programmes effectively reduce stress, anxiety, and depression during pregnancy. It could be beneficial to include MF in prenatal care and for healthcare personnel related to pregnant women to be trained in this technique in order to be able to provide it.

The findings show that this approach can alleviate symptoms related to mood disorders, anxiety, and stress and should therefore be considered as a means of preventing or reducing psychological distress.

## Figures and Tables

**Table 1:** Summary of the articles included in the review.

Author(s); Year and Country of Publication; [Paper No.]	Study Design	Sample Size and Mean Age of Participants	Description of MF Intervention	Anxiety		Depression		Stress	
				Mean (SD) Pre-	Mean (SD) Post-	Mean (SD) Pre-	Mean (SD) Post-	Mean (SD) Pre-	Mean (SD) Post-
Abatemarco et al., 2021 USA [29]	Experimental study MF-based intervention for pregnant women at high risk for preterm birth, considering stress, anxiety, and depression, together with race (African American) and low socioeconomic status.	Sample: n = 35 Age 18–24 years: n = 7 (20.0%) Age 25–35 years; n = 24 (68.6%) Age ≥ 36 years: n = 4 (11.4%) Completed first part of the study (Post 1) n = 27 (77%) Completed seven months postpartum (Post 2) n = 19 (54%)	Six two-hour sessions (once weekly) during pregnancy, focusing on stress, anxiety, MF, and depression. Study variables evaluated 2 months post-intervention and during the postpartum period (7 months post-intervention). Questionnaires used to evaluate the study variables: − Perceived Stress Scale PSS − Pregnancy-Specific Anxiety Scale PSA − Spielberger’s Trait Anxiety Inventory STAI − Edinburgh Postpartum Depression Scale EPDS − Five-Facet Mindfulness Questionnaire FFMQ	Baseline n = 35 13.0 (0.53)	POST 1 n = 27 11.2 (0.57) POST 2 n = 19 10.2 (0.74)	Baseline n = 35 11.3 (0.98)	POST 1 n = 27 1.4 (1.16) POST 2 n = 19 8.2 (1.2)	Baseline n = 35 20.7 (1.0)	POST 1 n = 27 16.5 (1.2) POST 2 n = 19 15.7 (1.3)
Agampodi et al., 2019 Sri Lanka [30]	Experimental study Incorporating an MF-based programme into prenatal care.	Sample size predetermined, not calculated n = 12–15 Final sample size: n = 12 Age: 18–30 years Characteristics of participants - No history of mental disorder with a psychotic component - Able to read and write Sinhalese - Gestational age <32 weeks	Eight sessions, of 2–3 h, once weekly. Semi-structured, anonymous, self-administered questionnaires were used to determine the cultural appropriateness, utility and feasibility of the programme. Overall goal: to promote mental well-being.	---	---	---	---	n = 12 All participants reported a change in how they responded to stressful situations, such as household and work tasks. Moreover, they worked more efficiently, achieving greater comfort and relaxation, in body and mind.	7 of the 8 women observed reduced stressors in their daily lives and gained a sense of calm. 3 of the 8 women felt they were better able to control their anger.
Baniaghil et al., 2022 Iran [31]	Randomised field study To determine the effect of MF-based group counselling on worries and stress for women during a first pregnancy.	114 women, never previously pregnant. Divided into two groups: Intervention group (n = 53) Age (mean ± SE) 26.21 ± 4.61 years Control group (n = 61) Age (mean ± SE) 25.52 ± 4.38 years Gestational age 12–20 weeks	For the intervention group, eight weekly sessions of 120–150 min) Data were compiled and groups formed using the Pregnancy Worries and Stress Questionnaire PSWQ Each intervention group completed the PWSQ at the end of the eighth session. Simultaneously, the control groups were asked over the phone to complete the questionnaire again on the same day or the day after the intervention groups’ last session. Based on the available data, all 96 participants in both the intervention and control groups completed the questionnaire upon con-clusion of the final session.	---	---	---	---	23.46 (13.03)	34.96 (15.88) Mean pregnancy stress and worry scores before and after MF-based group counselling improved by 11 units.
Dimidjian et al., 2016 USA [32]	Randomised clinical trial Applying MF-based cognitive therapy versus usual care to prevent the recurrence of perinatal depression.	86 pregnant women with a history of depression. Divided into two groups: Intervention group: 43 women underwent MF-Based Cognitive Therapy (MBCT) for Perinatal Depression Age: 30.98 (SE: 4.08) years 26 women completed the intervention Control group: 43 women received usual treatment (UT) 36 women completed the intervention Age: 28.72 (SE: 5.50) years	8 sessions, of which 7 were practical. Each 6-day week was considered a session. Total duration: 42 days. To consider the intervention completed, at least 4 sessions must be attended. The first session consisted of an SCID-I/P interview (diagnosis and statistics of mental disorders) and a DSM-IV interview (to evaluate the presence of personality disorders). The possible recurrence of depression was assessed by Longitudinal Interval Follow-up Evaluation, a semi-structured interview, consistent with DSM-IV-TR diagnostic criteria at 8 weeks and 1 month prepartum and 1 and 6 months postpartum, to assess recurrence status after the intervention. The Edinburgh Postpartum Depression Scale (EPDS) was used to assess the severity of depression symptoms. This evaluation was performed at baseline, immediately before randomisation, midway through, and immediately following the intervention, at each session of MBCT-PD, and monthly for the remainder of pregnancy and up to six months postpartum 8-item self-reported Client Satisfaction Questionnaire completed at the 8-week and 6-month postpartum assessments.	---	---	First, the power of the statistical test comparing MBCT-PD and UT was calculated. For this population sample (n = 86), the dropout rate was 19.8% during follow-up, the statistical power obtained was 71.4% and 81.4% for the two- and one-tailed tests, respectively For a 30% difference in the relapse rate, the statistical power was 84.3%.	MF group Relapses at 6 months: 18.4% Control group Relapses at 6 months: 50.2% For the MF group, the risk of relapse was 30% lower. According to CSQ-8, 90% of the MF participants were committed and highly satisfied.	---	---
Epel et al., 2019 USA [33]	Quasi-experimental study Stress can provoke excessive weight gain. Analysis of MF-based stress reduction (Mindful Moms Training, MMT).	n = 215 Divided into two groups: Control group (n = 105) Age (SD) = 28.0 (6.0) years Completed the intervention: n = 90 Intervention group (n = 110) Age (SD) = 27.8 (5.7) years Completed the intervention: n= 95	8 weekly sessions of 2 h of Mindful Moms Training (MMT) 2 booster telephone sessions, 1 postpartum group session Participants completed the following questionnaires on psychological distress, eating patterns, and exercise at baseline and 8 weeks post-intervention. - Cohen’s Perceived Stress Scale - Patient Health Questionnaire PHQ-9 - Pregnancy-Related Anxiety Scale PRA - Acceptance and Action Questionnaire-II - Dutch Eating Behavior Questionnaire - Yale Food Addiction Scale - Stanford Brief Activity Survey - US Adult Food Security Survey Module	Control group Baseline 2.1 (0.7) MF Group Baseline 2.1 (0.6)	Control group 2.0 (0.6) MF Group 2.0 (0.7)	Control group Baseline 6.8 (4.9) MF Group Baseline 7.6 (5.6) Depression (β = −2.00; 95% CI = −3.39, 0.62)	Control group 6.1 (4.5) MF Group 4.5 (3.7) *	Control group Baseline 18.4 (6.6) MF Group Baseline 19.1 (6.6) Perceived stress (β = −2.01, 95% CI = −3.93, −0.09)	Control group 17.0 (7.4) MF Group 15.6 (5.8) * * Sample size varied due to missing data, ranging from 167 to 170 for the final sample with complete data for each measure. * *p* < 0.05
Goodman et al., 2014 USA [34]	Experimental study Analysis of CALM Pregnancy programme for pregnant women with generalised anxiety disorder, high levels of anxiety, or symptoms of worry.	n = 24 of whom 23 attended an average of 6.96 sessions) 21 women (87.5%) attended at least 6 of the 8 sessions Mean age (SD) = 33.5 (4.4) years Range: 27–45 years	8 weekly group sessions of 2 h (groups of 6–12 women) 30–40 min of home practice daily during the intervention. Instruments used: − Penn State Worry Questionnaire PSWQ − GAD-7 − Patient Health Questionnaire-9 PHQ-9 − MINI-International Neuropsychiatric Interview − Beck Anxiety Inventory − Beck Depression Inventory—Second Edition BDI-II − Self-Compassion Scale − Mindfulness Attention Awareness Scale	Baseline n = 11 (47.8%)	n = 7 (63.6%) (recovered) n = 2 (18.2%) (Significantly improved)	Baseline n = 23 (100%)	Post-MF n = 11 (47.8%) (recovered) n = 5 (21.7%) (significantly improved)	---	---
Guardino et al., 2014 USA [35]	Controlled randomised clinical trial (experimental study) MF-based intervention for women experiencing high levels of perceived stress and anxiety during pregnancy.	n = 47 Divided into two groups: Intervention group n = 24 Mindful awareness practical classes Completed the intervention: n = 20 Control group (n = 23) Completed the intervention: n = 20 Mean age of participants: 33.13 years (SD = 4.79)	Six weeks of 2 h MF classes at the Mindful Institute of the Semel Institute at UCLA. The following online questionnaires were completed immediately post-intervention (Time 1) and 6 weeks later (Time 2). Five-Facet Mindfulness Questionnaire FFMQ Perceived Stress Scale PSS Pregnancy Specific Anxiety PSA Pregnancy-Related Anxiety Scale PRA Spielberger’s Trait Anxiety Inventory STAI The participants in the control group were given a booklet each trimester of pregnancy with information on childbirth, postpartum feeding, and infant care.	MF Group Baseline PSA 11.63 (2.96) Control group PSA 10.70 (2.79) MF Group Baseline STAI 45.69 (7.64) Control group Baseline STAI 44.37 (10.98)	MF Group POST 1 PSA 7.65 (1.73) POST 2 PSA 9.20 (2.35) Control group POST 1 PSA 8.95 (3.00) Control group POST 2 PSA 9.00 (2.23) MF Group POST 1 STAI 39.47 (6.27) POST 2 STAI 38.11 (8.78) Control group POST 1 STAI 37.35 (11.51) POST 2 STAI 36.19 (10.84)	---	---	MF Group Baseline PSS 41.81 (6.00) Control group Baseline PSS 39.91 (8.55)	MF Group POST 1 PSS 37.30 (5.38) POST 2 PSS 36.17 (5.90) Control group POST 1 PSS 35.80 (8.01) POST 2 PSS 37.42 (7.27)
Kalmbach et al., 2023 USA [36]	Controlled randomised clinical trial Study conducted to determine whether cognitive behavioural therapy (the Perinatal Understanding of Mindful Awareness for Sleep (PUMAS) programme) is effective in combating prenatal insomnia, depression, and cognitive arousal.	One group: n= 12 11 PUMAS patients (91.7%) completed the 6 sessions Age = 22 to 36 years (30.33 ± 4.23) (Mean ± SD)	6 weekly individual telemedicine (i.e., video) sessions of 60 min. PUMAS The results were evaluated by: − Insomnia Severity Index ISI − Edinburgh Postnatal Depression Scale EPDS − Pre-Sleep Arousal Scale PSAS-C—nocturnal cognitive arousal). Nocturnal thought focused on the perinatal period was assessed using an item attached to the PSAS-C. − Glasgow Sleep Effort Scale GSES − Consumer Report Treatment Satisfaction Scale CRTSS Self-efficacy in MF meditation was assessed after the intervention.	---	---	pre PUMAS Baseline EPDS 8.67 ± 5.33	post- PUMAS EPDS 3.42 ± 2.75	---	---
Kundarti et al., 2023 Indonesia [37]	Quasi-experimental study (randomised control study) MF-based intervention to measure and reduce levels of anxiety and cortisol during pregnancy.	n = 70 Divided into two groups: Intervention group (n = 35) Mean age: 23.80 ± 2.96 years Control group n = 35 Mean age: 25.31 ± 3.03 years	Eight 2 h MF sessions, once weekly. The PASS questionnaire was completed. A DBC blood cortisol test with competitive ELISA I was performed, after obtaining informed consent. Data on anxiety and blood cortisol were collected at baseline and after 8 weeks (post-test).	MF Group Baseline 34.77 (17.26) Control group 39.23 (20.56)	MF Group POST 12.83 (8.29) Control group 30.69 (16.39)	---	---	---	---
Nejad et al., 2021 Iran [38]	Randomised clinical trial To evaluate how an MF-based stress reduction programme influences stress, anxiety, and depression resulting from an unplanned pregnancy.	n = 60 with unplanned pregnancy Divided into two groups: Intervention group (n = 30) Mean age: 28.93 ± 5.62 years Control group n = 30 Mean age: 29.30 ± 6.32 years	8 MF-based stress reduction sessions (2 h/session, once weekly), plus home practice and recorded audio. Results were assessed using the Depression, Anxiety and Stress Scale DASS-21, at baseline and after the 8 sessions.	MF Group Baseline 13.20 (7.05) Control group 12.20 (6.06)	MF Group POST 3.33 (2.98) Control group 13.6 (6.54)	MF Group Baseline 19.80 (16.13) Control group 18.8 (7.95)	MF Group POST 4.6 (5.09) Control group 17.86 (9.9)	MF Group Baseline 23.86 (0.859) Control group 25.40 (8.07)	MF Group Baseline 8.86 (5.45) Control group 23.86 (8.74)
Pan et al., 2023 Taiwan [39]	Longitudinal randomised clinical trial Testing the effect of a perinatal MF programme on stress, anxiety, depression, and bonding in women with a perinatal mood and anxiety disorder.	n = 102 Divided into two groups: Intervention group (n = 51) Completed intervention n = 33 Mean age (SD): 33.52 ± 4.91 years Control group n = 51 Completed intervention n = 33 Mean age (SD): 32.88 ± 3.90 years	8-week prenatal MF programme, with one 2 h session each week. Results were assessed using the following instruments: − Edinburgh Postnatal Depression Scale EPDS − Perceived Stress Scale-10 PSS-10 − Postpartum Bonding Questionnaire PBQ − Pregnancy-Related Thoughts Scale PRT Efficacy of the intervention was assessed: − Before randomisation (T0) − Post-intervention (T1) − At 36 weeks of gestation (T2) − At 2 months after delivery (T3) − At 4 months after delivery (T4) Depression and stress were measured at T0, T1, T2, T3, and T4. Anxiety was measured at T0, T1, and T2.	MF Group Baseline 24.21 Control group 22.69	MF Group POST 17.53 T1 (B = 0.84. *p* < 0.001. ES = 0.74; large effect) T2 (B = 0.85. *p* < 0.001; ES = 0.47; moderate effect) Control group 18.05	MF Group Baseline 12.88 (2.99) Control group 13.70 (3.78)	MF Group Baseline 9.12 (T1) 9.18 (T2) 7.18 (T3) 9.43 (T4) This decrease was significant for the MF group at T1 (B = −0.69. *p* < 0.001. ES = 0.52): T2 (B = 0.73. *p* < 0.001. ES = 0.22). (no effect) Control group 11.67 (T1) 10.36 (T2) 9.79 (T3) 10.55 (T4)	MF Group Baseline 18.45 (4.91) Control group 18.97 (3.78)	MF Group Baseline 14.18 (T1) 14.85 (T2) 13.88 (T3) 14.40 (T4) (slight) The decrease was significant for the MF group at T1 (B = −0.26, *p* < 0.001, ES = 0.53) and T2 (B = 0.62, *p* < 0.001, ES = 0.29). After delivery, the z scores for PSS fell in the MF group (B = 0.62, *p* < 0.001; B = −0.66, *p* < 0.001) and the effect size was small to moderate at T3 and T4 (ES = 0.56; 0.21) Control group 16.76 (T1) 16.12 (T2) 16.64 (T3) 15.57 (T4)
Zhang et al., 2023 China [40]	Randomised clinical trial Conducted to determine the effectiveness of a guided digital self-help MF-based intervention in reducing maternal psychological distress and improving the child’s neuropsychological performance.	n = 160 Randomly divided into two groups: Digital GSH-MBI group n = 80 Mean age (SD) 30.36 (4.65) years Completed intervention: n = 69 11 did not complete Control group n = 80 Mean age (SD) 30.21 (3.93) years Completed intervention: n = 66 14 did not complete Dropout rate: 25/160, 15.6% Mean age (SD) 30 (4.29) years	6 weeks/6 modules. Guided digital self-help MF-based intervention, using 10–20 m video modules via WeChat mini-program. On the first day of each week, a video was screened. On the remaining 6 days of each week, the participants had formal audio-based practices and assignments focused on mindful breathing and body scanning, plus informal MF practices in everyday life or 3 min space-to-breathe exercises. Outcomes were assessed at 6 weeks and at 6 months postpartum, using the following scales and questionnaires. − Depression and anxiety: EPDS and GAD-7. − Early Childhood Temperament Questionnaire (neuropsychological development). − Child Behavior Questionnaire (revised) Assessment schedule: T1: Baseline (12–20 weeks’ gestation) T2: Immediately after the intervention (approx. 20–28 weeks’ gestation) T3: Before birth (36–37 weeks’ gestation) T4: At 6 weeks postpartum T5: At 3 months postpartum T6: At 6 months postpartum. The effect of the intervention was analysed using generalised estimating equations.	Control group Baseline 5.80 (3.14) (T1) 5.61 (3.04) (T2) 6.18 (3.83) (T3) 7.31 (4.49) (T4) 5.90 (4.71) (T5) 5.90 (4.76) (T6) R/C Pregnancy 21.88 (4.64) (T1) 23.15 (5.55) (T2) 24.22 (5.77) (T3)	MF group Post 5.56 (2.61) (T1) 3.14 (2.74) (T2) 3.32 (3.19) (T3) 4.49 (3.63) (T4) 4.34 (3.31) (T5) 3.75 (3.28) (T6) Wald χ2_5_ = 24.7; *p* < 0.001) R/C Pregnancy 22.61 (4.53) (T1) 19.47 (4.03) (T2) 19.41 (4.98) (T3) Wald χ2^2^ = 46.5; *p* < 0.001)	Control group Baseline 9.43 (3.26) (T1) 7.86 (5.07) (T2) 8.60 (5.58) (T3) 9.25 (6.34) (T4) 8.27 (6.31) (T5) 8.45 (6.53) (T6)	MF group Baseline 8.91 (3.54) (T1) 5.21 (4.46) (T2) 4.48 (4.22) (T3) 5.81 (5.27) (T4) 5.25 (4.47) (T5) 5.54 (5.44) (T6) (Wald χ2_5_ =20.6; *p* = 0.001)	---	---
Zhang et al. (2021) China [41]	Randomised clinical trial Conducted to examine the effectiveness of an MF-based intervention in reducing prenatal stress compared to participation in a health education (HE) group.	n = 108 Divided into two groups: Intervention group: n = 54 Control group n = 54 Mean age (SD) 28.85 (3.60) years Range: 21–42 years	30 m weekly sessions for 4 weeks via WeChat plus 30–45 m daily MF practice. Depression and anxiety were assessed using EPDS and GAD-7 in the last week and in the final 2 weeks. Perceived stress was assessed with the Perceived Stress Scale-4 PSS-4 The severity of fatigue was assessed using the Fatigue Severity Scale FSS The effect of the intervention was analysed using generalised estimating equations. Assessment schedule: T1: Baseline T2: Immediately after the intervention T3: 15 weeks after the intervention	Control group 5.63 (3.06) (T1) 5.98 (3.74) (T2) 5.90 (3.37) (T3)	MF Group 5.73 (2.66) (T1) 4.56 (2.74) (T2) 4.83 (1.78) (T3) The main effects of study group, time, and group-time interaction for anxiety were not significant: (Wald v2 = 3.46. *p* = 0.063; Wald v2 = 1.49. *p* = 0.475; Wald v2 ¼5.16. *p* = 0.076.	Control group 10.35 (2.64) (T1) 9.54 (3.90) (T2) 10.14 (4.33) (T3)	MF Group 9.88 (3.21) (T1) 7.17 (3.81) (T2) 7.39 (3.29) (T3) Main significant effect for the group: (Wald v2 = 5.00. *p* = 0.005) and for time (Wald v2 ¼22.85. *p* < 0.001). and one non-significant effect for the group-time interaction (Wald v2 = 6.01. *p* = 0.049) for depression.	Control group 5.86 (2.15) (T1) 6.59 (5.53) (T2) 6.95 (2.77) (T3)	MF Group 5.51 (2.25) (T1) 4.38 (2.45) (T2) 3.14 (2.07) (T3) Main significant effect for the group (Wald v2 = 30.47, *p* < 0.001) and a main non-significant effect for time (Wald v2 = 2.40, *p* = 0.301), and a significant effect for the group—perceived stress interaction (Wald v2 ¼26.94, *p* < 0.001).

## Data Availability

Not applicable.

## Appendix A

**Table A1:** MMAT critical appraisal.

Study	Q1	Q2	Q3	Q4	Q5
Abatemarco et al., 2021 [29] Agampodi et al., 2019 [30]	Can’t tell	Yes	Yes	Can’t tell	Yes
Agampodi et al., 2019 [30]	Yes	Yes	Can’t tell	Yes	Yes
Baniaghil et al., 2022 [31]	Yes	Yes	Yes	Yes	Yes
Dimidjiam et al., 2016 [32]	Yes	Yes	Yes	Yes	Yes
Epel et al., 2019 [33]	Yes	Yes	Yes	Yes	Yes
Goodman et al., 2014 [34]	Can’t tell	Yes	Yes	Yes	Yes
Guardinoa et al., 2014 [35]	Yes	Yes	Yes	Can’t tell	Yes
Kalmbach et al., 2023 [36]	Can’t tell	Yes	Yes	Can’t tell	Yes
Kumdarti et al., 2023 [37]	Yes	Yes	Yes	Can’t tell	Yes
Nejad et al., 2021 [38]	Yes	Yes	Yes	Can’t tell	Yes
Pan et al., 2023 [39]	Yes	Yes	Yes	Yes	Yes
Zhang et al., 2023 [40]	Yes	Yes	Yes	Yes	Yes
Zhang et al., 2021 [41]	Yes	Yes	Yes	Yes	Yes
